# Binding of the transcription factor MYC2-like to the ABRE of the *OsCYP2* promoter enhances salt tolerance in *Oryza sativa*

**DOI:** 10.1371/journal.pone.0276075

**Published:** 2022-10-14

**Authors:** Hongbo Liu, Peng Cui, Bingxin Zhang, Jinbo Zhu, Cui Liu, Qingyang Li

**Affiliations:** Key Laboratory for Quality Improvement of Agricultural Products of Zhejiang Province, College of Advanced Agricultural Sciences, Zhejiang A & F University, Hangzhou, China; Institute of Crop Science, CHINA

## Abstract

Cyclophilins, a type of peptidyl-prolyl *cis*-*trans* isomerase, function as important molecular chaperones in a series of biological processes. However, the expression pattern and signal transduction pathway of cyclophilins are still unclear. Here, we showed that the promoter of *OsCYP2* could function as a tissue-specific promoter by GUS staining. Moreover, we found that the promoter sequence contained not only core elements but also inducible elements. Then, the ABA-responsive element was used for cDNA library screening, and the transcription factor MYC2-like was identified by a yeast one-hybrid assay and confirmed through an electrophoretic mobility shift assay. Furthermore, the relative expression showed that *MYC2-like* was induced by abscisic acid. In addition, *MYC2-like* overexpression enhanced salt tolerance in transformants and partially restored the *cyp2*-RNAi line. In summary, we explored a novel transcriptional signal mediated by MYC2-like, a potential regulator of salt stress-related physiological processes in rice.

## Introduction

Salt, drought, cold and toxic metals in the soil are major environmental factors that affect the geographical distribution of plants in nature and limit plant productivity in agriculture. Abiotic stresses are often unfavorable or stressful for plant growth and development [1]. Conversely, crops undergo a series of morphological, physiological, biochemical and molecular changes to adapt to these stress conditions during their lifetimes [2, 3]. The development of crops with elevated levels of salt tolerance is therefore highly desirable. How plants sense stress signals and adapt to adverse environments are fundamental biological questions. To date, there have been many constraints in the complexity and low genetic variance of quantitative traits during previous genetic modifications [4, 5]. With the development of genomics, transcriptomics and proteomics resources, a large array of stress-responsive transcription factors has been identified with functional research in crops [6–8]. Most importantly, promoter structure analysis and yeast one-hybrid assays have been applied as efficient technologies in transcription factor studies [9–11].

Basic/helix-loop-helix (bHLH) proteins are a superfamily of transcription factors that bind as homodimers and/or heterodimers to a consensus hexanucleotide sequence called the E-box (5’-CANNTG-3’). Among the different types of E-boxes, the most common palindromic G-box (5’-CACGTG-3’) provides a core consensus site for the recognition of bHLH transcription factors [12]. In addition, flanking nucleotides outside of the hexanucleotide core sequence and loop residue in bHLH transcription factors could affect the binding specificity [13, 14]. To date, a series of MYC2-like transcription factors containing bHLH domains have been well characterized in crops as crucial regulatory components in diverse abiotic and biotic stress responses [15, 16]. More importantly, there is some evidence that bHLH transcription factors mediate hormone signaling to regulate crop biological processes [17, 18].

Therefore, novel stress-inducible genes/proteins can help to reveal the unknown molecular mechanisms of complex traits in abiotic stress tolerance. In a previous study, *cyclophilin 2* (*CYP2*), as a molecular chaperone, was shown to confer salt tolerance in rice by modulating the activities of antioxidant enzymes at the translational level [19]. Moreover, we hope to elucidate the precise regulatory mechanism underlying the key transcription factors involved in this signaling pathway. Here, we present the expression pattern of *OsCYP2* and characterize its transcriptional signal mediated by MYC2-like, a potential regulator of salt stress-related physiological processes via abscisic acid (ABA) signaling in rice.

## Materials and methods

### Cloning and analysis of the *OsCYP2* promoter

Wild type seeds (*Oryza sativa* L. cv. Aichi-ashahi) were germinated with distilled water in a dark incubator at 30 °C and cultured at 32 °C/28 °C with a photoperiod of 16 h/8 h (light/dark). The total DNA of seedlings was extracted from the wild type according to the modified CTAB method. An upstream 1156 bp fragment of *OsCYP2* was amplified by specific primers (S1 Table in S1 File) and then used to substitute the promoter that can drive *GUS* in pCAMBIA1301 to construct a *pOsCYP2*:*GUS* plasmid (S1 Fig in S1 File). The *cis* elements of the promoter were predicted by PLACE [20].

### Histochemical GUS assay

The p*OsCYP2*:*GUS* vector was transformed into the wild type by *Agrobacterium tumefaciens* strain EHA105 according to a previously described method with minor modification [21]. GUS activity was conducted with various tissues (seed, leaf, stem and root) of wild type and transformants via histochemical staining by 0.5 mg/mL X-gluc (5-bromo-4-chloro-3-indolyl-β-D-glucuronide), which contained 80 mM phosphate buffer, 0.5 mM K_3_Fe(CN)_6_, 0.5 mM K_4_Fe(CN)_6_, 0.1% Triton X-100, and one drop of N,N-dimethyl formamide. All the samples were incubated at 37 °C for 24 hr and rinsed for 30 min with a series of 50%, 75%, and 100% ethanol. Then, these samples were soaked in 75% ethanol for analysis and photography.

### Construction of bait yeast strain and testing for aureobasidin A (AbA) expression

Based on *cis* elements of the promoter, the ABA responsive element (ABRE) target sequence (p62) was used as a bait, and the mutant sequence (p63) was used as a negative control. Two antiparallel oligonucleotide sequences (S1 Table in S1 File) of ABRE *cis* elements with *Hind*III and *Xho*I overhanging sticky ends were synthesized, and then, the oligonucleotides were annealed and ligated into the linearized pAbAi vector. Recombinant plasmids were linearized through *Bst*BI and transformed into Y1H to generate bait reporter yeast strains. The minimal inhibitory concentration of AbA was used to bait the reporter yeast strain.

### Yeast one-hybrid assay

The preconstructed cDNA library was transformed into the Y1H bait strain (pBait-AbAi) using the Yeastmaker Transformation System 2 (Clontech, Cat. No. 630439) [22]. The transformation reaction was spread on SD/-Leu plates with minimal inhibitory concentration of AbA. The candidate clones were restreaked onto SD/-Leu/AbA at least three times to generate a single colony. To eliminate duplicate clones, we conducted yeast colony PCR with Matchmaker Insert Check PCR Mix 2 (Clontech, Cat. No. 630497). The prey vectors that showed a single band in yeast colony PCR were isolated by an Easy Yeast Plasmid Isolation Kit (Clontech, Cat. No. 630467) and sequenced using T7 primers. For verification of positive interactions, each prey should be transformed into bait and mutant bait strains on selective media with side-by-side positive and negative controls.

### Electrophoretic mobility shift assay

The transcription factor MYC2-like was cloned into pET28a for electrophoretic mobility shift assays (EMSAs). The recombinant vector was transformed into *Escherichia coli* BL21 competent cells, and the fusion protein was purified with a Magnehis Protein Purification Kit (Promega, Cat. No. V8500). The samples were analyzed by 12% SDS-PAGE and western blots with His tag antibody conjugated to horseradish peroxidase. The oligonucleotides containing three tandem copies of the elements are listed in S1 Table in S1 File and labeled by a Biotin 3’ End DNA Labeling Kit (Thermo, Cat. No. 89818). EMSAs were conducted using a Light Shift Chemiluminescent EMSA Kit (Thermo Fisher Scientific, Cat. No. 20148) according to the manufacturer’s instructions.

### Transcript level of *MYC2-like* under salt and ABA induction

Wild type seedlings were cultivated as previously described. Samples were treated with 150 mM NaCl and 50 μM ABA. The transcript level of *MYC2-like* was determined by quantitative RT-PCR after treatment for 2 hr, 12 hr and 24 hr. The primers for *MYC2-like* are listed in S1 Table in S1 File. Three replicates were used for each sample and assayed according to reference [23].

### Construction of *MYC2-like* overexpression transformants in wild-type and *cyp2*-RNAi lines

The recombinant vector p*1300-Ubiquitin-myc2-like* was constructed and then transformed into the wild-type and *cyp2*-RNAi line L3-61 [22] through the *A*. *tumefaciens* strain EHA105 to create overexpression transformants. Plant transformation was performed according to a previously described method with minor modification [21]. Ten-day-old independent transformants of *MYC2-like* overexpression in wild-type and *cyp2*-RNAi lines were identified by quantitative RT-PCR.

### Physiological and biochemical assays of transformants

For evaluation of the salt response of transformants, 10-day-old seedlings of the wild type, *MYC2-like* overexpression in wild type, *MYC2-like* overexpression in *cyp2*-RNAi and *cyp2*-RNAi groups were treated with 150 mM NaCl for 24 hr in three replications. Fresh leaves (0.5 g) were ground and homogenized in 10 ml of precooled potassium phosphate buffer (pH 7.0). The mixture was centrifuged and used for the determination of various antioxidant enzymes. The physiological and biochemical indices were measured according to reference [24] and included superoxide dismutase (SOD), peroxidase (POD), catalase (CAT), malondialdehyde (MDA) and proline. In addition, fresh leaves (0.5 g) were digested with H_2_SO_4_ and H_2_O_2_ and then used to analyze the sodium and potassium ion contents through atomic absorption spectrophotometry [25].

### Statistical analysis

Statistical analysis of the results presented here are the means of three replications. Treatment means were compared by the analysis of variance and using the least significant difference test at the P≤0.05 level of significance.

## Results

### Promoter cloning and *cis* element analysis

The recombinant vector that contained an upstream 1156 bp fragment of *OsCYP2* was identified by double digestion with *Bam*HI and *Bgl*II (S2 Fig in S1 File). Furthermore, data regarding predicted *cis*-acting regulatory elements are listed in S2 Table in S1 File. This promoter not only contains core elements (TATA, CAAT and GATA box) but also contains inducible elements (ABRE and MYBR) that respond to abiotic stresses.

### GUS staining and transcript level analysis of *OsCYP2*

**Fig 1:**
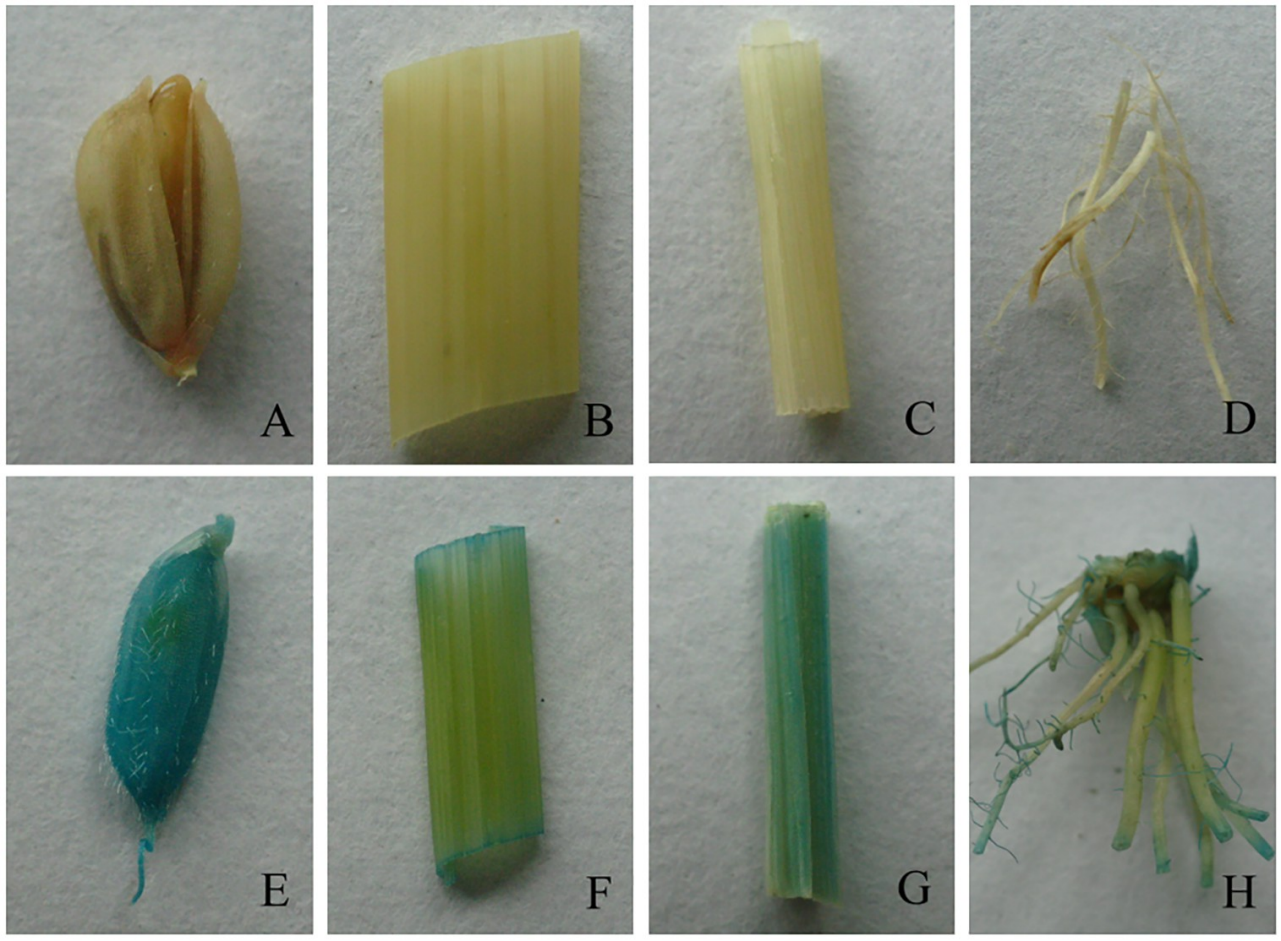
Staining showed expression patterns of *OsCYP2* indicated by the *GUS* reporter gene which was driven by the promoter of *OsCYP2*. A-D: seed, leaf, stem and root of wild type; E-H: seed, leaf, stem and root of the p*OsCYP2*:*GUS* transgenic plants.

In GUS staining, the results of the histochemical assay showed that four tissues (seed, leaf, stem and root) of transformants were stained blue, which were induced by the promoter of *OsCYP2* (Fig 1E–1H), but not in the wild type (Fig 1A–1D). In particular, GUS activity was higher in seeds and stems than in other tissues, indicating that the promoter of *OsCYP2* could function as a tissue-specific promoter.

### Screening a cDNA library and rescuing a prey plasmid

**Fig 2:**
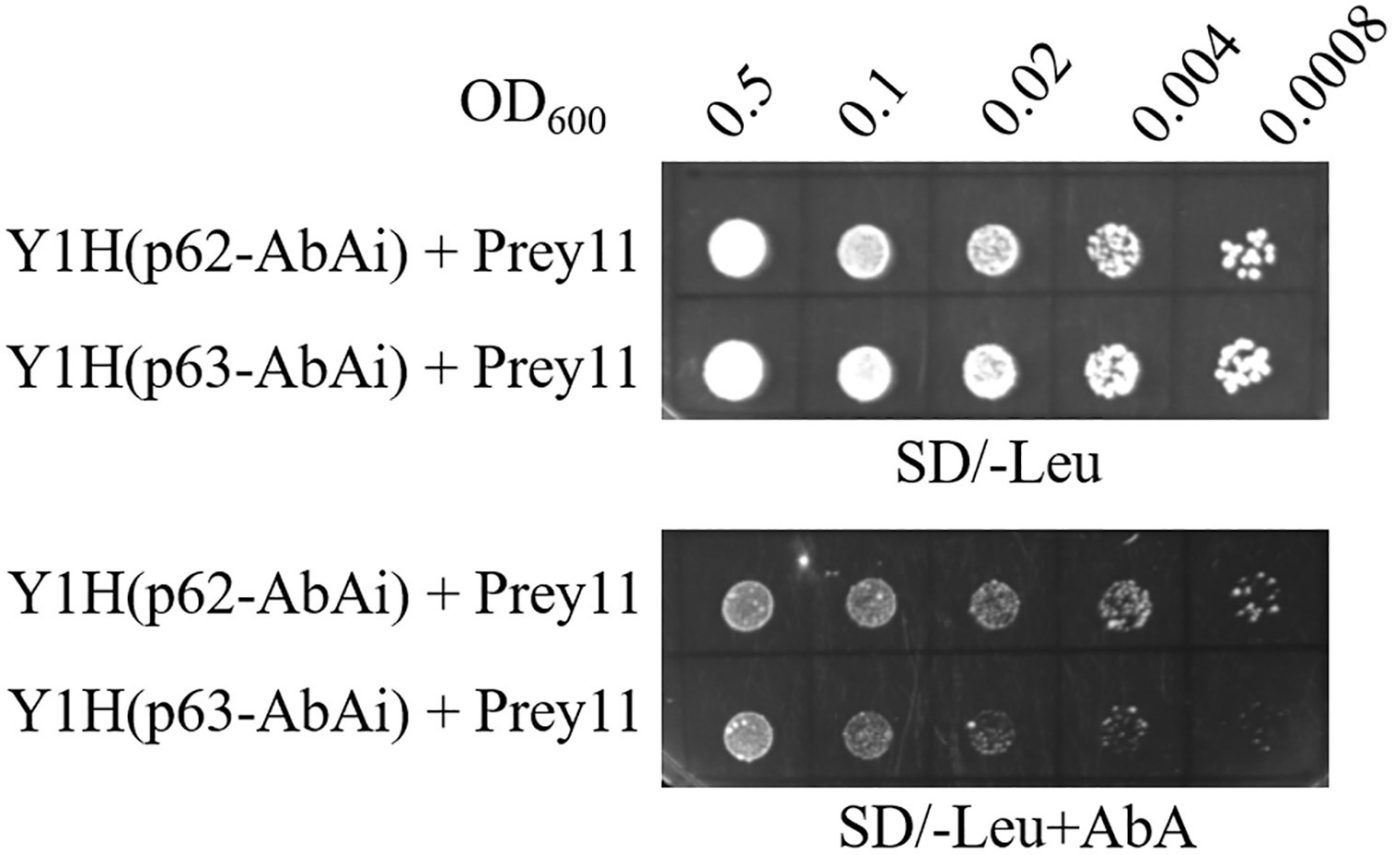
Cotransformation and serial dilution (1:5) with a initial optical density OD_600_) of 0.5 on selective media to verify the interaction of candidate transcription factor (No. 11). SD/-Leu: synthetically defined medium with dropout leucine, SD/-Leu+AbA: synthetically defined medium with dropout leucine and plus 750 μg/L Aureobasidin A; Y1H (p62-AbAi): bait strain; Y1H (p63-AbAi): mutant bait strain.

Four types of Y1H strains (p62-AbAi, p63-AbAi, p53-AbAi, and pAbAi) were constructed and used to determine the minimal inhibitory concentration of AbA. The results showed that neither the positive control Y1H (p53-AbAi) nor the negative control Y1H (pAbAi) can grow on SD/-Ura with 150–750 μg/L AbA. The effective inhibitory effects were validated at 750 μg/L for Y1H [p62-AbAi] and Y1H [p63-AbAi] for cDNA library screening (S3 Fig in S1 File). For a further step, sixteen colonies were obtained on SD/-Leu/AbA plates by library scale transformation of the Y1H [p62-AbAi] strain. Among them, twelve colonies showed good growth after streaking three times on SD/-Leu/AbA media plates (S4 Fig in S1 File). The results of yeast colony PCR showed that 10 colonies (Nos. 3, 5–8, 9, 11, 12, 14–16) had one band, except for No. 12 (two bands) (S5 Fig in S1 File). In distinguishing positive from false interactions, the results showed that candidate prey (No. 11) could grow on selective media plates (Fig 2), indicating a genuine interaction compared to other false interactions (S6 Fig in S1 File). Furthermore, a MYC2-like protein (Gene ID: 4349484) was identified by sequencing and BLAST.

### MYC2-like protein binds to ABREs *in vitro*

**Fig 3:**
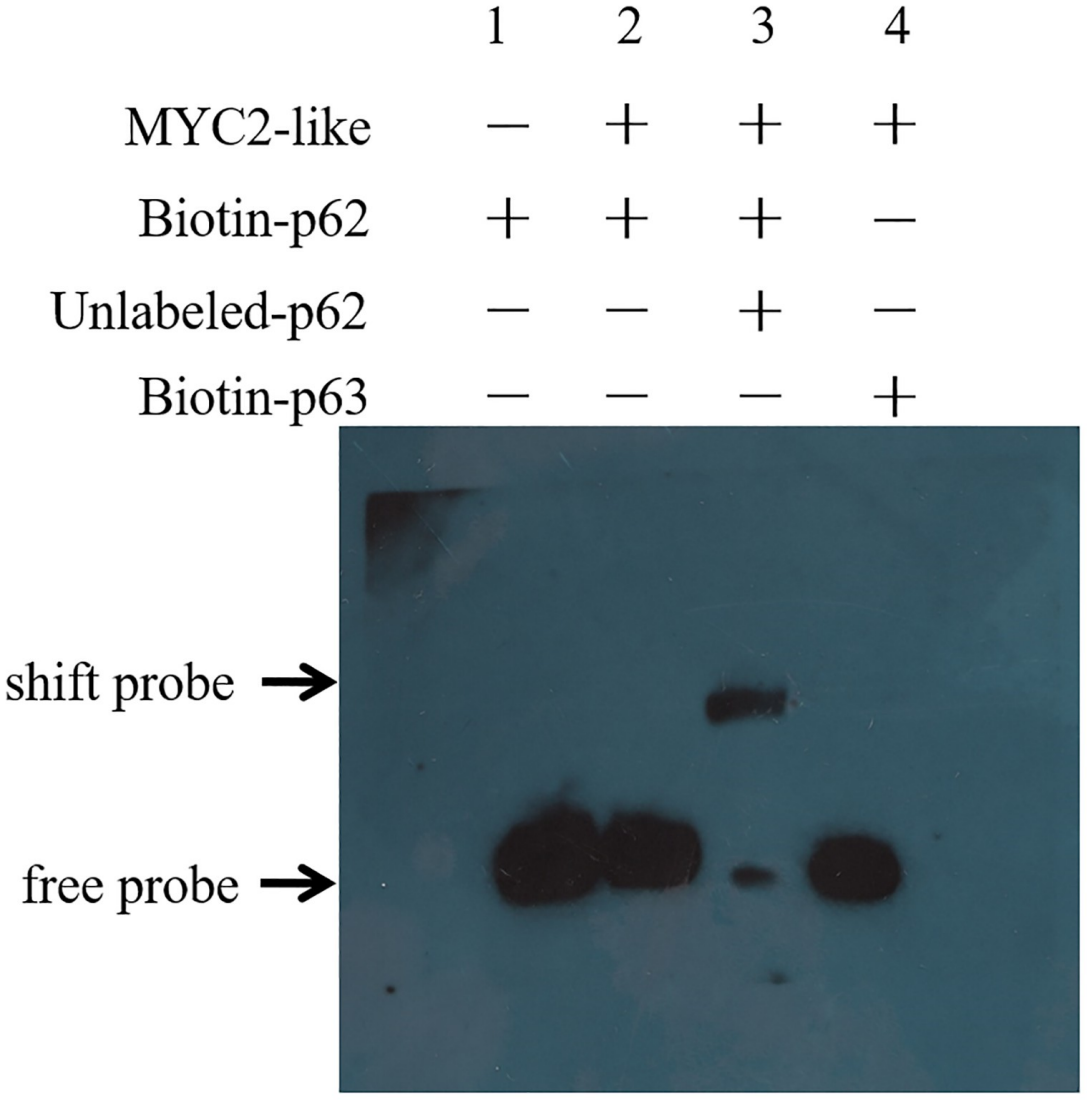
MYC2-like protein bind to the ABRE element by EMSA assays. 1: no MYC2-like protein for biotin labeled probe (p62) to bind, establishes the position of an unshifted probe band; 2: the MYC2-like protein was incubated with the biotin labeled probe (p62); 3: 200-fold excesses of unlabeled probes were used for competition; 4: biotin labeled mutant probe (p63) were used as a negative control.

To investigate the *in vitro* interaction between MYC2-like protein and the ABRE, we identified a His-tagged fusion MYC2-like protein although SDS-PAGE and western blotting (S7 Fig in S1 File). The results of EMSAs showed that MYC2-like protein could directly bind to biotin-labeled probes with a shift band by comparison to unconjugated and biotin-mutated probes (Fig 3). In addition, the signal shift observed can be prevented by competition from 200-fold molar excess unlabeled probes.

### *MYC2-like* was induced by salt and ABA

**Fig 4:**
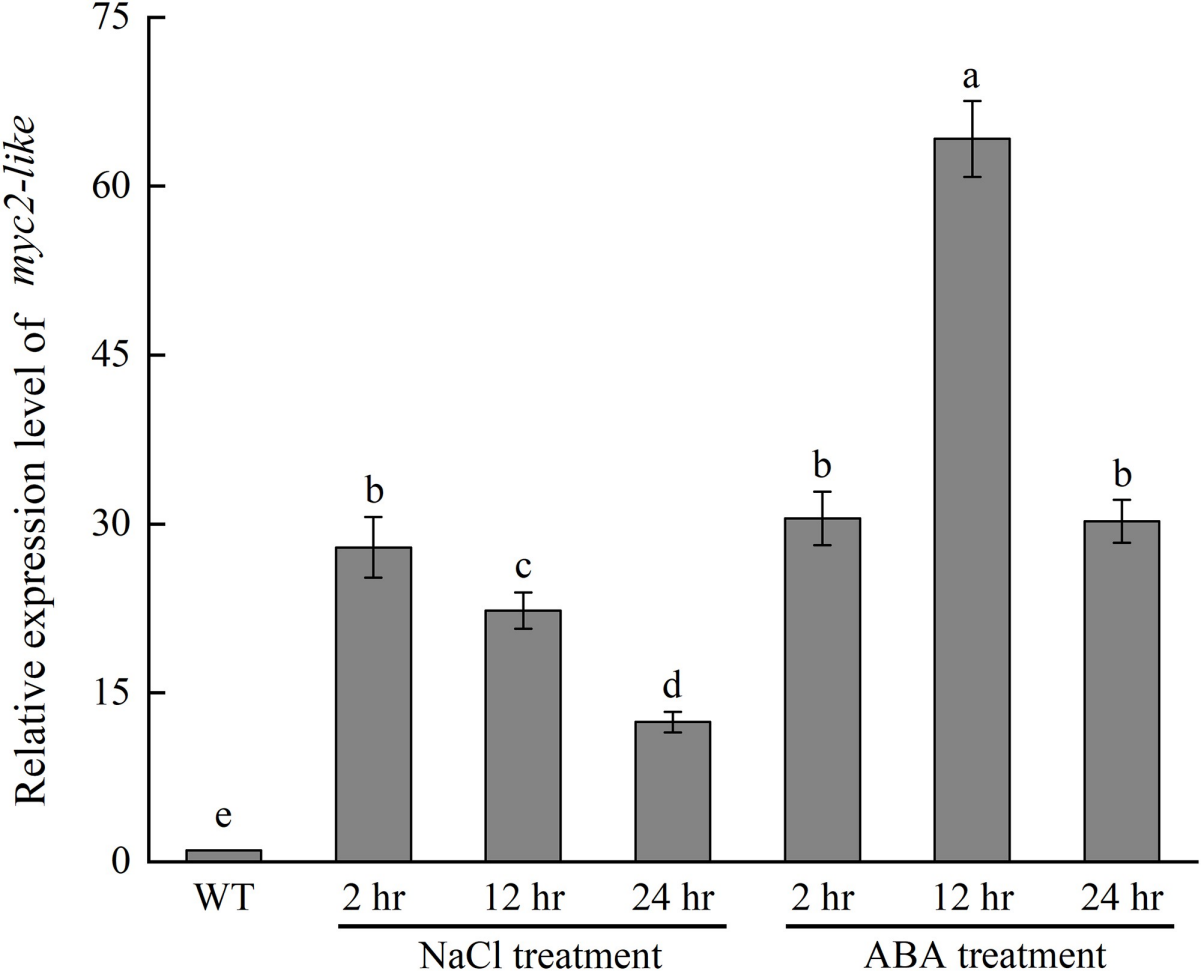
Relative transcript level of the *MYC2-like* induced by salt and ABA. Error bars represent the standard deviation.

To better understand the expression pattern of *MYC2-like* in response to salt and ABA, we detected the transcript level with a series of treatments (Fig 4). The expression of *MYC2-like* could be induced both by salt and ABA at 2 h, 12 h and 24 h. Furthermore, the expression demonstrated a different trend. For NaCl treatment, the transcript level gradually decreased over time from 2–24 hr; however, the maximal expression occurred at 12 hr under ABA treatment.

### Phenotypic characterization and physiological responses to salt stress of transformants

**Fig 5:**
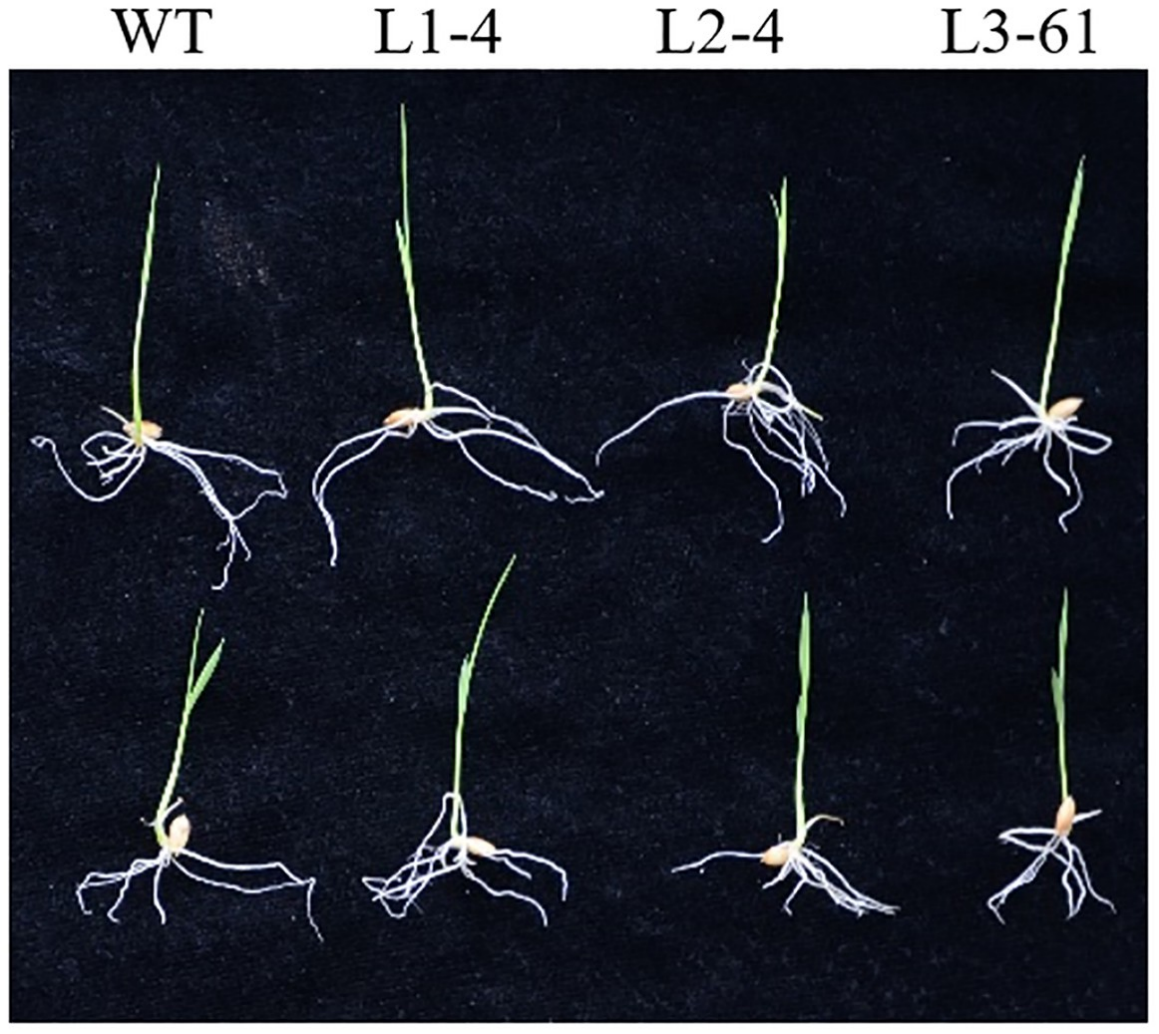
Phenotype of wild type, *MYC2-like* overexpression (L1), *MYC2-like* overexpression in *cyp2*-RNAi (L2) and *cyp2*-RNAi (L3) under salt stress.

**Fig 6:**
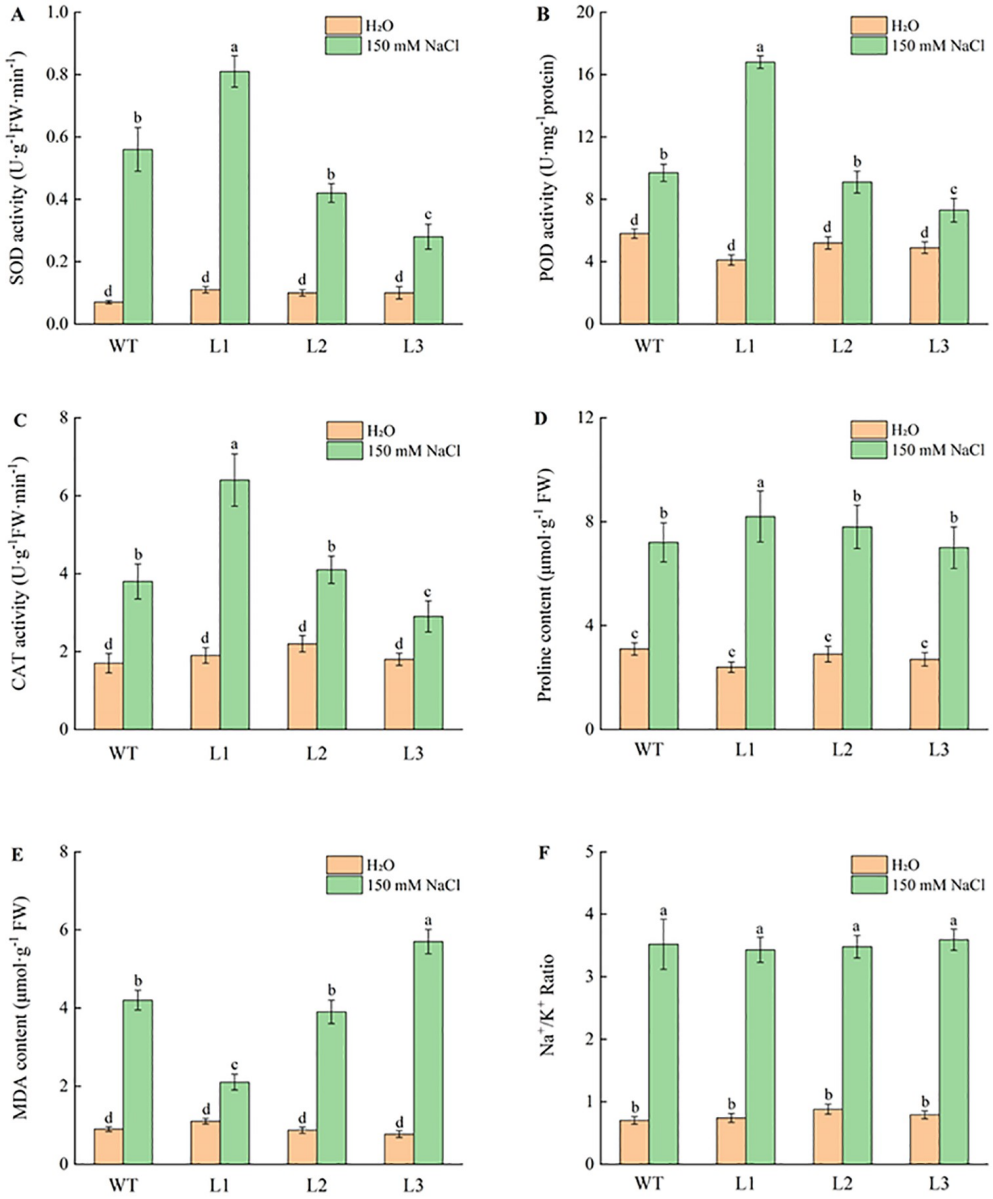
Comparison of antioxidative enzymes (SOD, POD, CAT), membrane lipid peroxidation index (MDA), osmolyte (proline), and Na^+^/K^+^ ratio among wild type and transformants under salt stress. Values are mean ± standard deviation followed by the same letter did not significantly differences at P≤0.05 according to least significant difference test.

Based on the results of qRT-PCR (S8 Fig in S1 File), three independent lines with *MYC2-like* overexpression in wild type and *MYC2-like* overexpression in *cyp2*-RNAi were selected for salt tolerance analysis. After treatment for 24 hr, the leaves of each line showed wilting, especially the wild type, and *cyp2*-RNAi (L3-61) exhibited even more sensitivity to salt stress (Fig 5). Obviously, *MYC2-like* overexpression (L1-4) resulted in better growth conditions, which implied that this gene partially restored the *cyp2*-RNAi (L2-4) phenotype under salt stress (Fig 5). The SOD, POD, and CAT activities in *MYC2-like* overexpression transformants were significantly increased by 44.6%, 73.2% and 68.4%, respectively, compared to that of the wild type (Fig 6A–6C). Similar results were exhibited in *cyp2*-RNAi and *MYC2-like* overexpression plants with the *cyp2*-RNAi background. In addition, the MDA and proline contents suggested that *MYC2-like* overexpression may result in better resistance to salt stress by reducing lipid peroxidation (Fig 6D and 6E). However, there were no significant differences in the Na^+^/K^+^ ratio among these lines under control conditions and NaCl treatment, suggesting that *MYC2-like* cannot adapt to salt stress selectively through Na^+^ and K^+^ uptake (Fig 6F).

## Discussion

An important feature of salt stress is that the hyperosmotic signal causes the accumulation of the phytohormone ABA, which in turn elicits many adaptive responses in crops. Therefore, crops have developed a series of alterations in physiology and biochemistry at the transcriptional and translational levels to offset the adverse effects that will allow them to avoid stress conditions [26]. There are two types of stress-responsive genes: functional and regulatory genes [27]. In a previous study, a cyclophilin chaperone named OsCYP2 was shown to promote resistance to abiotic stresses, especially salt stress, when overexpressed [19]. However, the transcriptional mechanism of this signaling pathway is unclear. In the present study, we aimed to analyze the expression pattern and promoter sequence of *OsCYP2* to provide more pivotal information to elucidate the mechanism of transcriptional regulation in response to salt stresses. A 1156 bp fragment upstream of *OsCYP2* was cloned and was shown to function as a tissue-specific promoter, which is especially efficient in induction in seeds and stems (S2 Fig in S1 File and Fig 1). It was implied that the different regulatory regions of the promoter may be responsible for tissue-specific expression [28].

*Cis* elements of promoters play an important role in the activation and suppression of gene expression in the transcriptional regulatory process, which depends on specific binding sequences for proteins [29, 30]. The ABA responsive element (ABRE, PyACGTGGC), including various types of DNA core sequences (G-box, CE3, hex3 and motif III), is the major binding site for ABA-regulated transcription factor targets [31]. The interaction between transcription factors and ABREs has been demonstrated to play a critical role in ABA-mediated responses [12, 31]. In a previous study, the expression of *OsCYP2* could be induced by several abiotic stresses and ABA [19]. Notably, 5 ABREs were found in the *OsCYP2* promoter sequence. Then, a MYC2-like transcription factor was identified to bind the ABRE by yeast one-hybrid and EMSAs (Figs 2 and 3).

There are two types of plant growth responses to salinity: the osmotic phase and ionic phase [32]. First, organic solutes accumulate in the cytosol and organelles at high salt concentrations to balance the osmotic pressure of the ions in the vacuole. Second, plant salt tolerance is mainly associated with low maintenance of the cytosolic Na^+^/K^+^ ratio [33, 34]. According to our results, *MYC2-like* overexpression could elevate the content of proline in transformants compared to the wild type in the presence of 150 mM NaCl, but there were no significant differences in the Na^+^/K^+^ ratio among these lines (Fig 6). However, *MYC2-like* overexpression transformants showed higher activity of antioxidative enzymes in reactive oxygen species scavenging. Therefore, we speculated that the *MYC2-like* gene increases resistance to salt stress by increasing the activity of antioxidative enzymes and post-translational regulation in transformed plants rather than selectively through Na^+^ and K^+^ uptake.

## Conclusions

To date, a clear understanding of the perception of salt and the signaling pathway has helped us to develop crop cultivars resistant to salinity and use salt-affected lands for productive cultivation. The identification and characterization of interactive transcription factors expressed under salt-responsive promoter elements could provide a promising approach for genetic breeding. In summary, our results indicated that OsCYP2 is involved in salt stress resistance through a novel transcriptional signal mediated by the MYC2-like transcription factor regulated in rice. More importantly, this molecule is a potential regulator that can provide a foundation in these sophisticated regulatory mechanisms to salt stress response and genetic improvement in rice.

## Supporting information

S1 File (DOCX)
