# Supplementary material for: Binding of the transcription factor MYC2-like to the ABRE of the OsCYP2 promoter enhances salt tolerance in Oryza sativa
Source: PLoS One. 2022 Oct 14;17(10):e0276075. doi: 10.1371/journal.pone.0276075 (PMC9565382; doi:10.1371/journal.pone.0276075)
Supplement: S1 File — (DOCX) [file pone.0276075.s001.docx]

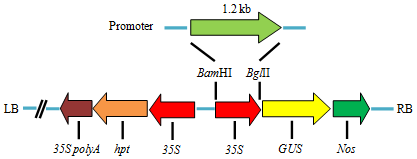


**S1 Fig. Schematic diagram of p*OsCYP2:GUS* based on the pCAMBIA1301 framework.**


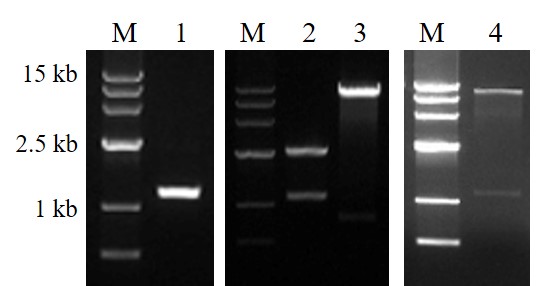


**S2 Fig.** **Construction of the p*OsCYP2:GUS* vector.** M: Wide Range 500-15000 marker; 1: fragment of promoter; 2 and 3: digested pMD19-T + promoter and pCAMBIA1301, respectively; 4: identification of the recombinant p*OsCYP2:GUS* vector.


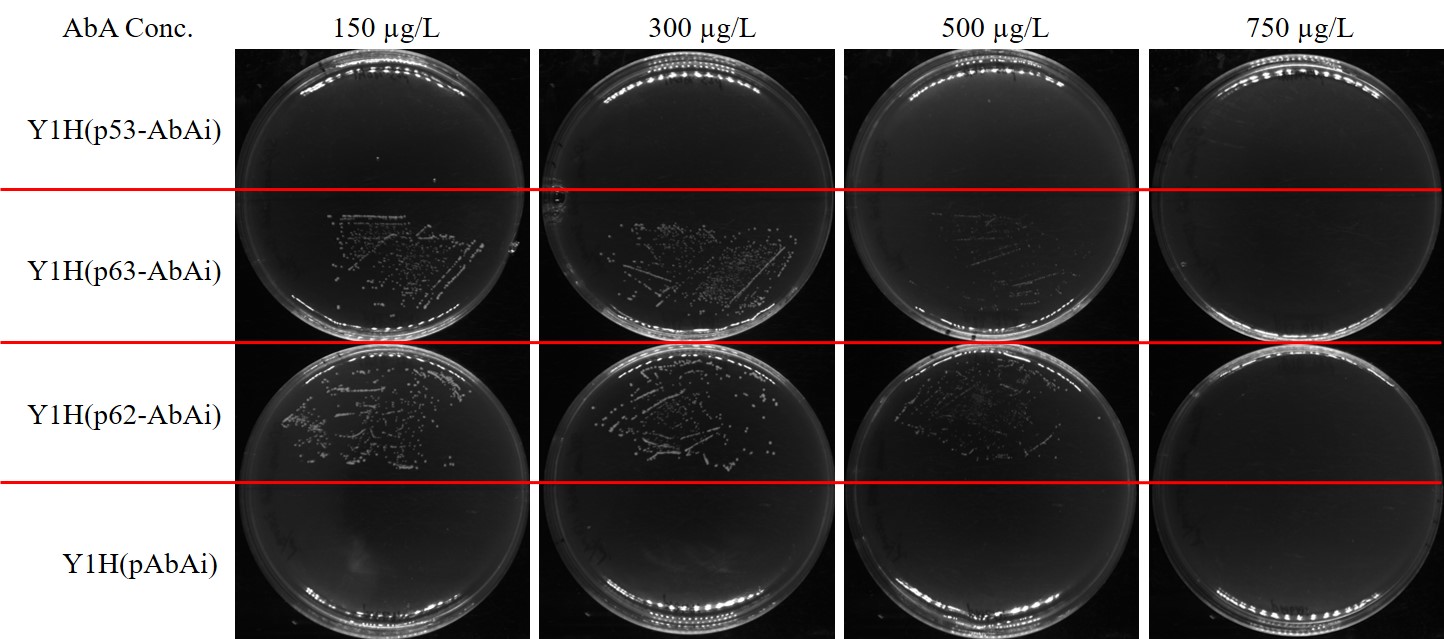


**S3 Fig. Determination of the minimal inhibitory concentration of AbA for the bait strain.** Y1H (p53-AbAi), Y1H (pAbAi), Y1H (p62-AbAi), and Y1H (p63-AbAi) represent positive, negative, bait and mutant bait strains respectively.


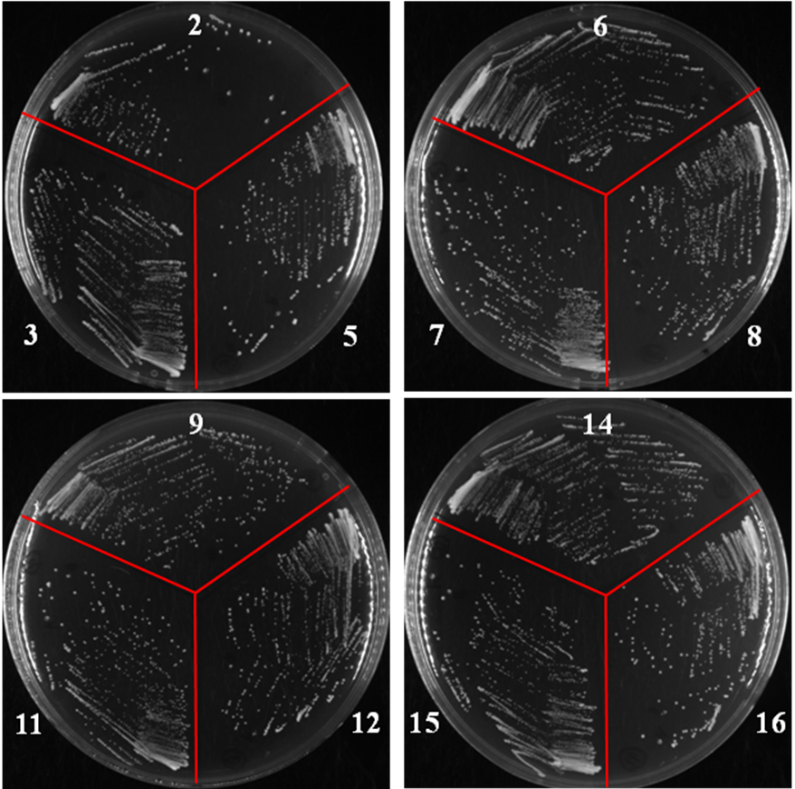


**S4 Fig. Partial colonies were restreaked onto SD/-Leu/AbA media plates.**


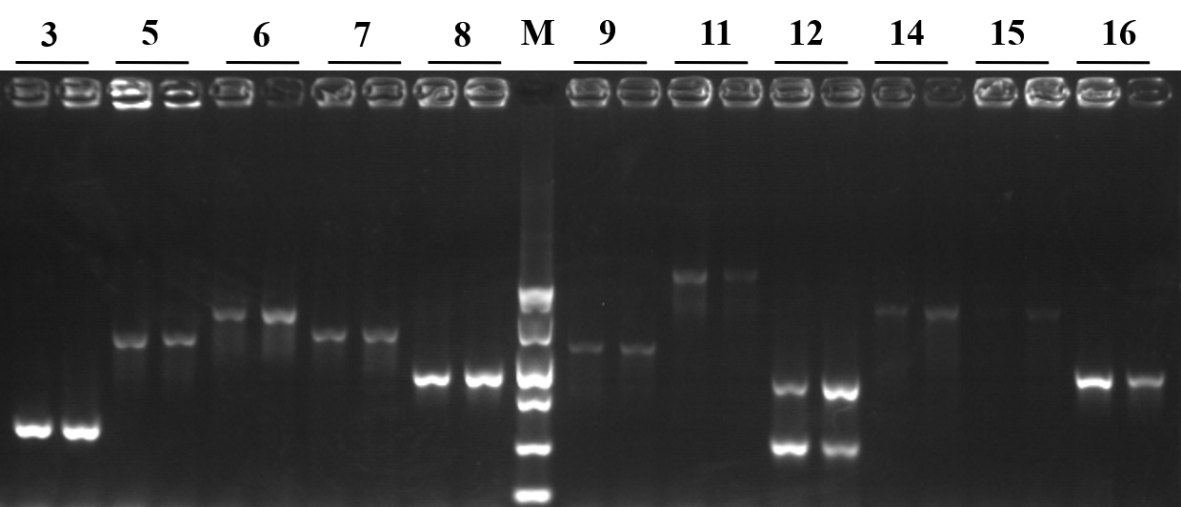


**S5 Fig. Detection of prey by yeast colony PCR.** Each prey using two independent colonies, M: DNA Marker F (200-2000).


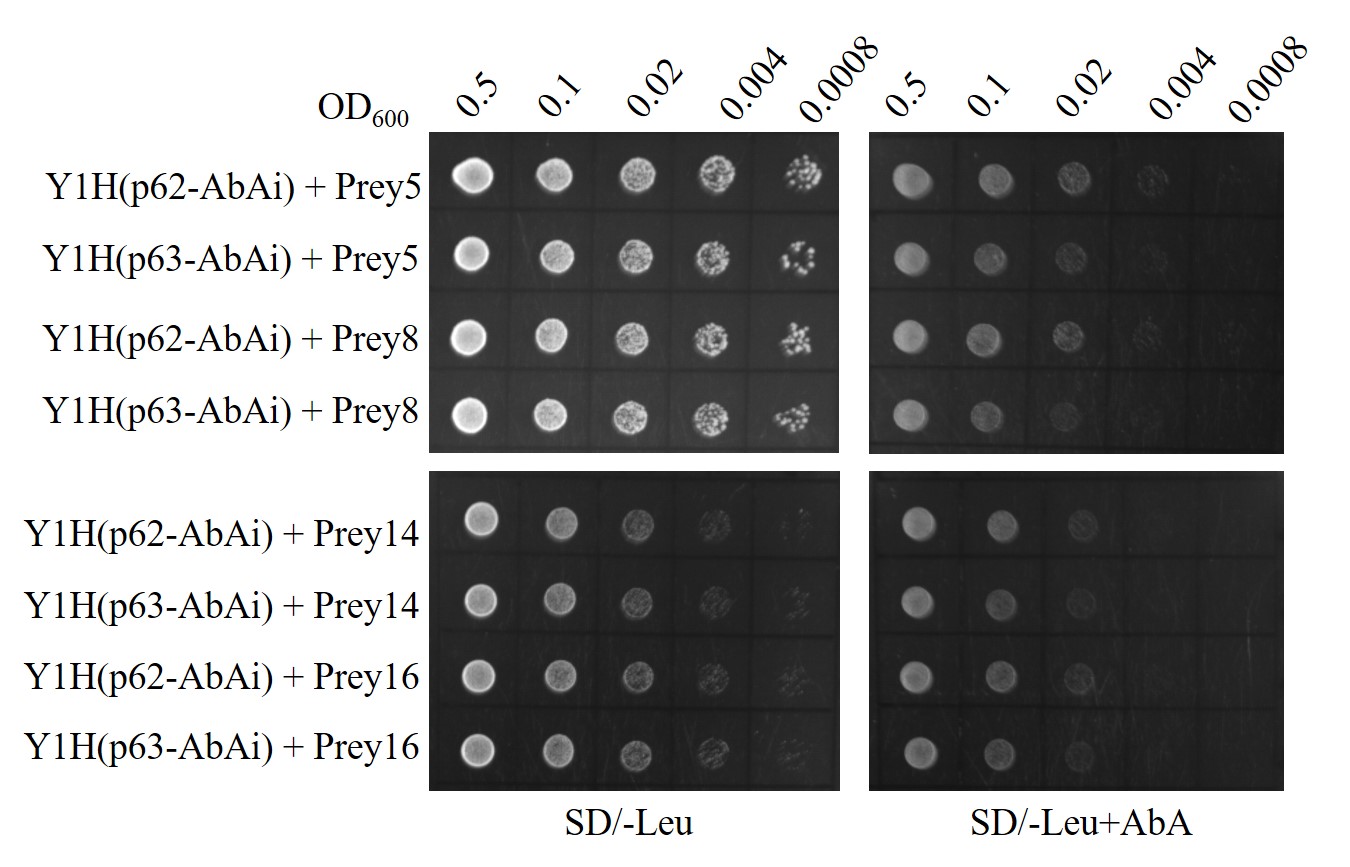


**S6 Fig. Cotransformation and serial dilution (1:5) with an initial optical density (OD_600_) of 0.5 on selective media to verify the interactions of candidate interaction transcription factors.** SD/-Leu: synthetically defined medium with dropout leucine, SD/-Leu+AbA: synthetically defined medium with dropout leucine and plus 750 μg/L aureobasidin A; Y1H (p62-AbAi): bait strain, Y1H (p63-AbAi): mutant bait strain.


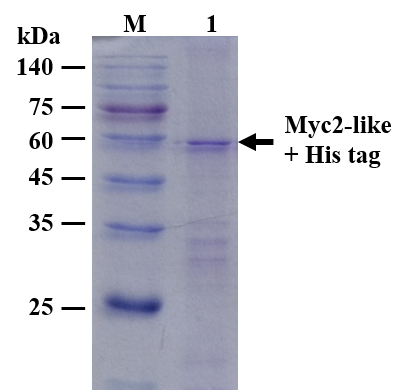


**S7 Fig****. Detection of recombinant MYC2-like with His tag by SDS‒PAGE and stained using Coomassie brilliant blue R-250.** M: prestained protein marker RM013; 1: myc2-like with His tag.


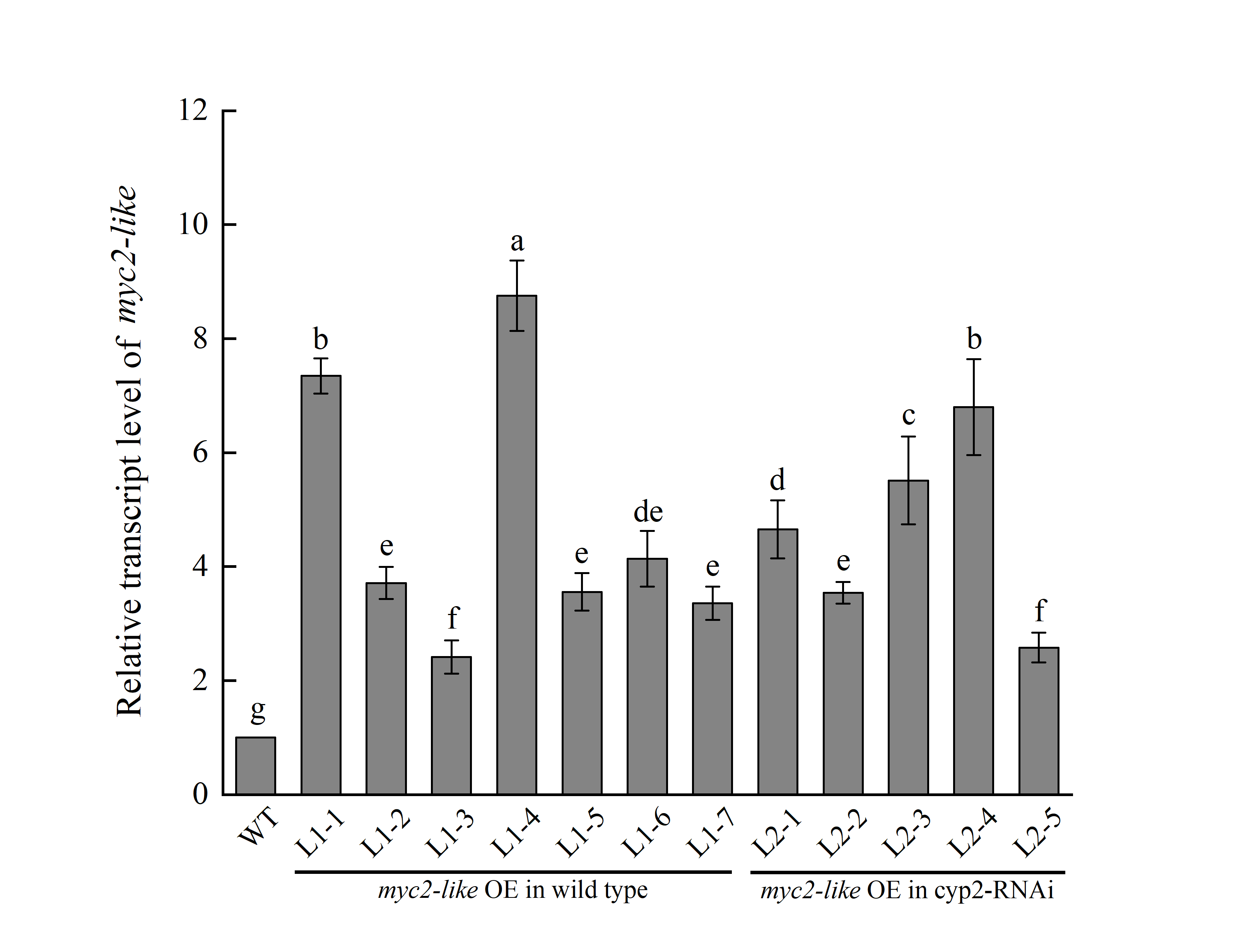


**S8 Fig. Relative transcript level of *MYC2-like* in the wild type, *MYC2-like* overexpression in wild type and *MYC2-like* overexpression in *cyp2* RNAi groups.** WT: wild type; L1-1, L1-2, L1-3, L1-4, L1-5, L1-6, L1-7: independent *MYC2-like* overexpression (OE) in wild type; L2-1, L2-2, L2-3, L2-4, L2-5: independent *MYC2-like* overexpression (OE) in *cyp2*-RNAi. Values represent means and those followed by different letters are significantly different (P≤0.05) by the least significant difference test. Error bars represent the standard deviation.

**S1 Table. Primers used in this study.**

| Primers | 5’ 3’ |
| --- | --- |
| OsCYP2 promoter FP | CGCGGATCCAACCGTAAAAAAGCCATGAT |
| OsCYP2 promoter RP | GGAAGATCTCTAGGGTTTTGCGAATTTC |
| p62 sense strand for Y1H | AGCTTCACGTGC |
| p62 antisense strand for Y1H | TCGAGCACGTGA |
| p63 sense strand for Y1H | AGCTTCTCGCGC |
| p63 antisense strand for Y1H | TCGAGCGCGAGA |
| p62 sense strand for EMSA | GGGTCACGTGCATC |
| p62 antisense strand for EMSA | GATGCACGTGACCC |
| p63 sense strand for EMSA | GGGTCTCGCGCATC |
| p63 antisense strand for EMSA | GATGCGCGAGACCC |
| Myc2-like FP for EMSA | CGGAATTCATGAACCTTTGGACGGACGA |
| Myc2-like RP for EMSA | CCAAGCTTCCGGGCGGCGGTGCCAGGCT |
| Myc2-like FP for overexpression | TCACCCGGGATGAACCTTTGGACGGACGA |
| Myc2-like RP for overexpression | TCACCCGGGTAGAGTTGAGTTACCGGGCG |
| Actin FP | GACCTTGCTGGGCGTGAT |
| Actin RP | GTCATAGTCCAGGGCGATGT |
| Myc2-like FP for qPCR | AGTGGTTCTTCCTCGTCTCC |
| Myc2-like RP for qPCR | AGGTTGAAGAGGGCGCGGAT |

Note: The underlines indicate restriction enzyme site

**S2 Table. Predicted *cis*-elements of the *OsCYP2* promoter.**

| Types | Elements | No. | Core sequence | Position ^c^ |
| --- | --- | --- | --- | --- |
| CE ^a^ | TATA box | 1 | TATAAAT | -736 (－) |
|  |  | 1 | TATATAA | -790 (＋) |
|  |  | 4 | TTATTT | -751 (＋), -813 (＋), -866 (＋), -1035 (－) |
|  |  | 1 | TATTTAA | -34 (＋) |
|  | CAAT box | 5 | CAAT | -204 (－), -669 (－), -711(－), -843 (－), -938 (－) |
|  | GATA box | 8 | GATA | -401 (－), -520 (－), -543 (－), -836 (＋), -985 (－), -1026 (－), -1050 (－), -1055 (＋) |
| IE ^b^ | ABRE | 2 | CACGTG | -86 (＋), -182 (＋) |
|  | MYBR | 3 | TAACCA | -110 (－), -142 (－), -153 (＋)， |
|  |  | 5 | CGGTT | -25 (－), -331 (－), -526 (－), -893 (＋), -1068 (－) |
|  |  | 3 | CCAACC | -45 (－), -337 (＋), -391 (＋) |

^a^ CE: core elements; ^b^ IE: inducible elements; ^c^ Position of the *cis*-elements relative to the putative transcription start site. Strands are indicated as: (＋), forward; (－), complement
